# Overall Performance Evaluation of Tubular Scraper Conveyors Using a TOPSIS-Based Multiattribute Decision-Making Method

**DOI:** 10.1155/2014/753080

**Published:** 2014-05-29

**Authors:** Yanping Yao, Ziming Kou, Wenjun Meng, Gang Han

**Affiliations:** ^1^Taiyuan University of Technology, Taiyuan 030024, China; ^2^Taiyuan University of Science and Technology, No. 66 Waliu Road, Wanbolin District, Taiyuan 030024, China

## Abstract

Properly evaluating the overall performance of tubular scraper conveyors (TSCs) can increase their overall efficiency and reduce economic investments, but such methods have rarely been studied. This study evaluated the overall performance of TSCs based on the technique for order of preference by similarity to ideal solution (TOPSIS). Three conveyors of the same type produced in the same factory were investigated. Their scraper space, material filling coefficient, and vibration coefficient of the traction components were evaluated. A mathematical model of the multiattribute decision matrix was constructed; a weighted judgment matrix was obtained using the DELPHI method. The linguistic positive-ideal solution (LPIS), the linguistic negative-ideal solution (LNIS), and the distance from each solution to the LPIS and the LNIS, that is, the approximation degrees, were calculated. The optimal solution was determined by ordering the approximation degrees for each solution. The TOPSIS-based results were compared with the measurement results provided by the manufacturer. The ordering result based on the three evaluated parameters was highly consistent with the result provided by the manufacturer. The TOPSIS-based method serves as a suitable evaluation tool for the overall performance of TSCs. It facilitates the optimal deployment of TSCs for industrial purposes.

## 1. Introduction

Tubular scraper conveyors are a new type of granular material conveyance equipment that utilizes flexible traction components. These conveyors have a number of advantages, including their compact construction, airtight conveyance, low energy consumption, low noise levels, good environmental performance, easy manipulation and maintenance, and long service life. In the production of nuclear fuel elements, the use of a tubular scraper conveyor eliminates the disadvantages from using either a screw conveyor or an air conveyor. A screw conveyor has a long conveying distance, a low conveying capacity, and severe machine-part wear. An air conveyor requires a substantial amount of power and multiple gas purification treatment devices. Moreover, a tubular scraper conveyor reduces radioactive environmental contamination by sealing the source materials in the equipment, thereby satisfying the requirements for technological developments in nuclear fuel element manufacturing [1].

Tubular scraper conveyors are primarily composed of traction components, a driving device, tensioner, pipeline system, feeding gate, and a discharge gate. The traction components (a chain or a wire rope equipped with disc scrapers) move inside the sealed-in pipeline, and scrapers convey the materials forward. The scraper space, material filling coefficient, and the dynamic load of the traction components affect the scraper force on the materials, stress upon the traction components, and the overall conveying efficiency. Both inaccurately specified parameters and a design that does not address all issues increase the driving power and accident rate of tubular scraper conveyors, which affects the overall efficiency of the conveyor and increases costs [2, 3]. Therefore, methods for properly evaluating the overall performance of tubular scraper conveyors have attracted substantial attention in related fields. Many scholars have attempted to improve the design of tubular scraper conveyors and have conducted numerous studies on this topic. These studies can be divided into two categories. The first category includes studies that derived theoretical stress-calculating models and obtained the basis for scraper space determination and running resistance calculation formulas through a dynamic mechanical analysis of the granular material in the bearing segment of the conveyor [4, 5]. The second category includes studies that used the comprehensive method of “dynamic optimization, intelligence optimization, visualization, and empirical examination” for dynamic and kinematic analyses of tubular scraper conveyor transfer systems to obtain an optimal dynamic design. The purpose of this latter category is to establish three-dimensional visualization models of tubular scraper conveyors for a finite element analysis of the machine parts and to evaluate, amend, and enhance the overall performance of the conveyor [6]. However, these studies have only focused on one or a few aspects of the comprehensive quality of the conveyor. Moreover, studies on the overall performance evaluation of tubular scraper conveyors are rare.

The overall performance evaluation of tubular scraper conveyors is a matter of multiattribute decision making. The overall performance of tubular scraper conveyors can be influenced by numerous uncertainty factors in actual production and life [7–9]. To date, the use of linguistic information based on numerical simulations has obtained remarkable achievements in solving this type of multiattribute decision problem in different fields, such as risk assessment, technology assessment, and achievement assessment [8]. The technique for order of preference by similarity to ideal solution (TOPSIS) is a member that has been widely used for such purposes. TOPSIS was first proposed by Hwang and Yoon [10]. This technique orders the solutions in the solution set  *X*  to the linguistic positive-ideal solution (LPIS) and the linguistic negative-ideal solution (LNIS) for multiattribute decision analyses to determine the optimal solution [11, 12]. The LPIS is the optimal virtual solution in the set  *X*  with the best attribute values in the decision matrix, whereas the LNIS is the worst virtual solution in the set with the worst attribute values in the matrix [13–17]. TOPSIS provides the overall condition for a comprehensive evaluation; therefore, this method has wide applicability. For example, TOPSIS has been used to evaluate health care quality, real estate investment site selection, the economic efficiency of enterprises, and coal mine projects [18]. Evaluating tubular scraper conveyors using the TOPSIS method assists in reducing the influence of design and subjective factors during conveyor use and in selecting the optimal solution. Therefore, TOPSIS may serve as a suitable method for the overall performance evaluation of tubular scraper conveyors.

In this study, we developed a TOPSIS-based model for evaluating the overall performance of tubular scraper conveyors considering multiple influential attributes. From a macroscopic perspective, a TOPSIS-based evaluation may satisfy a large range of customer requirements for the overall quality and performance of tubular scraper conveyors.

## 2. Data and Methods

### 2.1. Data

The scraper space (*t*), material filling coefficient (*η*), and the vibration coefficient of the traction components (*k*) are the major factors that affect the overall performance of tubular scraper conveyors. The scraper space directly affects the force transfer of the scrapers during the conveying process. Moreover, the material filling coefficient affects the conveying efficiency; the vibration of the traction components increases the relative motion between materials, which causes the motion to become unstable and increases the power consumption and noise of the conveyor.

The type of tubular scraper conveyor investigated in this study was the Tcca-200 (Jiaozuo Xinheng Heavy Industry Machine Co., Ltd., China). The company provided the coefficients for three factors that affect the overall performance of the conveyors. The design schemes of the three conveyors (A, B, and C) are summarized in Table 1.

In this study, the conveyed material was flour. The primary parameters consisted of a conveying volume of 40 m^3^/h, horizontal length of 20 m, and a hoisting height of 10 m.

### 2.2. Theoretical Background of TOPSIS

The TOPSIS method uses linguistic information based on numerical simulations to solve multiattribute decision problems. It is able to make full use of raw data with little information loss and therefore serves as an effective method for multiattribute decision making [7, 19]. Its basic operational principles are as follows. If there are *m* targets, each having *n* attributes [20], then the mathematical model of the multiattribute decision problem can be defined as [21–28]

(1) $$\begin{array}{c} S=\left\{s_{ij}\;|\;i=1,2\ldots ,m,\;\;j=1,2\ldots ,n\right\}, \end{array}$$

where *s*_*ij*_ is the evaluated value of the  *j*th attribute of the  *i*th target. The decision matrix *X* is given by

(2) $$\begin{array}{c} X=\left|\begin{array}{cccc} x_{11} & x_{12} & \cdots & x_{1n}\\ x_{21} & x_{22} & \cdots & x_{2n}\\ \vdots & \vdots & \vdots & \vdots \\ x_{m1} & x_{m2} & \cdots & x_{mn} \end{array}\right|. \end{array}$$

The counting backward method was used in this study. The low-optimization index *x*_*ij*_  (*i* = 1,2 …, *m*, *j* = 1,2 …, *n*) was transformed into the high-optimization index *x*_*ij*_′  (*i* = 1,2 …, *m*, *j* = 1,2 …, *n*) based on *x*_*ij*_′ = 1/*x*_*ij*_. The post-co trending decision matrix *X*′ is defined as follows:

(3) $$\begin{array}{c} X^{\prime }=\left|\begin{array}{cccc} x_{11}^{\prime } & x_{12}^{\prime } & \cdots & x_{1n}^{\prime }\\ x_{21}^{\prime } & x_{22}^{\prime } & \cdots & x_{2n}^{\prime }\\ \vdots & \vdots & \vdots & \vdots \\ x_{m1}^{\prime } & x_{m2}^{\prime } & \cdots & x_{mn}^{\prime } \end{array}\right|. \end{array}$$

The information-weighted matrix (*W*) of the attributes was obtained using the DELPHI expert group method [29]. The weighted judgment matrix (*P*) can be defined by

(4) P=|x11′x12′⋯x1n′x21′x22′⋯x2n′⋮⋮⋮⋮xm1′xm2′⋯xmn′|·W=|x11′x12′⋯x1n′x21′x22′⋯x2n′⋮⋮⋮⋮xm1′xm2′⋯xmn′||ω10⋯00ω2⋯0⋮⋮⋮⋮00⋯ωn|=|p11p12⋯p1np21p22⋯p2n⋮⋮⋮⋮pm1pm2⋯pmn|.

The matrices of the raw data after the same trending were normalized; the corresponding matrices were established based on the following index transformation formulas:

(5) $$\begin{array}{c} q_{ij}=\frac{x_{ij}}{\sqrt{\sum_{i=1}^{m}x_{ij}^{2}}},\\ q_{ij}^{\prime }=\frac{x_{ij}^{\prime }}{\sqrt{\sum_{i=1}^{m}{\left(x_{ij}^{\prime }\right)}^{2}}},\\ Q=\left|\begin{array}{cccc} q_{11} & q_{12} & \cdots & q_{1n}\\ q_{21} & q_{22} & \cdots & q_{2n}\\ \vdots & \vdots & \vdots & \vdots \\ q_{m1} & q_{m2} & \cdots & q_{mn} \end{array}\right|. \end{array}$$

Based on *Q*, the most and least optimal vectors, namely, the most optimal and least optimal solutions among the finite solutions, were defined as follows:

(6) $$\begin{array}{c} Q^{+}=\left(q_{i1}^{+},q_{i2}^{+},\ldots ,q_{in}^{+}\right),\\ Q^{-}=\left(q_{i1}^{-},q_{i2}^{-},\ldots ,q_{in}^{-}\right). \end{array}$$

The distances between each index value of the evaluated target and that of the optimal solution (*L*_*i*_^+^) and between each index value of the evaluated target and that of the least optimal solution (*L*_*i*_^−^) were calculated based on the following formulas:

(7) $$\begin{array}{c} L_{i}^{+}=\sqrt{\overset{n}{\underset{j=1}{\sum }}{\left(q_{ij}^{+}-q_{ij}\right)}^{2}},\\ L_{i}^{-}=\sqrt{\overset{n}{\underset{j=1}{\sum }}{\left({q_{ij}}^{-}-q_{ij}\right)}^{2}}. \end{array}$$

Furthermore, the approximation degree of each evaluated target to the optimal solution (*y*_*i*_) was calculated based on the following formula:

(8) $$\begin{array}{c} y_{i}=\frac{L_{i}^{-}}{L_{i}^{+}+L_{i}^{-}}, \end{array}$$

where *y*_*i*_ ∈ [0,1]. A value of *y*_*i*_ close to 1 indicates that the evaluated target is a degree closer to the optimal level, whereas the evaluated target is a degree closer to the least optimal level when *y*_*i*_ is close to 0. The evaluated targets were ordered according to the obtained *y*_*i*_ values: the overall performance of the evaluated target increases as *y*_*i*_ increases.

### 2.3. TOPSIS-Based Example Calculation

To obtain the TOPSIS-based results, the overall performances of the three tubular scraper conveyors of the same type were evaluated based on the weights of the scraper space (*t*), material filling coefficient (*η*), and the vibration coefficient of the traction components (*k*) when the overall performance reached optimal levels (Table 1).

There were three targets, and each target had three attributes. Based on (1), the mathematical model of the multiattribute decision problem can be expressed as follows:

(9) $$\begin{array}{c} S=\left\{s_{ij}\;|\;i=1,2,3,\;\;j=1,2,3\right\}. \end{array}$$

The decision matrix was found to be

(10) $$\begin{array}{c} X=\left|\begin{array}{ccc} 2.7 & 3.1 & 4.2\\ 2.6 & 3.0 & 4.4\\ 2.5 & 3.2 & 4.3 \end{array}\right|. \end{array}$$

Moreover, the post-same trending decision matrix *X*′ was calculated to be

(11) $$\begin{array}{c} X^{\prime }=\left|\begin{array}{ccc} 0.3704 & 0.3226 & 0.2381\\ 0.3846 & 0.3333 & 0.2273\\ 0.4 & 0.3725 & 0.2326 \end{array}\right|. \end{array}$$

The information-weighted matrix (*W*) was obtained using the DELPHI expert group method; the weighted judgment matrix (*P*) was determined to be

(12) P=X′W=|0.37040.32260.23810.38460.33330.22730.40.37250.2326||100010001|=|0.37040.32260.23810.38460.33330.22730.40.37250.2326|.

The post-same trending matrices of the raw data were normalized, and the corresponding matrix was established to be

(13) $$\begin{array}{c} Q=\left|\begin{array}{ccc} 0.5551 & 0.5767 & 0.5908\\ 0.5765 & 0.5960 & 0.5639\\ 0.5996 & 0.5587 & 0.5770 \end{array}\right|. \end{array}$$

The optimal and least optimal value vectors that were obtained from  *Q*  were found to be, respectively,

(14) $$\begin{array}{c} Q^{+}=\left(q_{i1}^{+},q_{i2}^{+},\ldots ,q_{in}^{+}\right)=\left(0.5996,0.5960,0.5908\right),\\ Q^{-}=\left(q_{i1}^{-},q_{i2}^{-},\ldots ,q_{in}^{-}\right)=\left(0.5551,0.5587,0.5639\right). \end{array}$$

Moreover, *L*_*i*_^+^, *L*_*i*_^−^, and *y*_*i*_ were subsequently calculated from the preceding results.

### 2.4. The Validation Method

Currently, the overall performance of tubular scraper conveyors is professionally evaluated based on their ability to satisfy the conveying capacity with the smallest conveyer power requirement and quietest traction components [10, 30]. To validate the results obtained from the TOPSIS method, we compared our results with the information supplied by the manufacturer. The operating noise and power of the three conveyors were measured by the manufacturer according to the following standards: (1) The National Standards of China (GB/T10596-2011): the operation of scraper conveyors should be stable and should not produce abnormal noise (5.1.4); the acoustic pressure level of the maximum noise 1 m away from the conveyor should be less than 85 dB during load running (5.1.11); (2) The Coal Industry Standards of the People's Republic of China (MT/T1097-2008): equipment should meet the requirements for efficacy, energy consumption, environmental protection, and sustainable development (1.0.10). The overall performances of the three conveyors were then ordered according to these measurement results (Table 2).

## 3. Results

### 3.1. TOPSIS-Based Results

In this study, we used the TOPSIS method to evaluate the overall performance of three tubular scraper conveyors of the same type that were manufactured by the same company. The results based on (7) and (8) are summarized in Table 3. Conveyor C exhibited the best overall performance, whereas conveyor A had the worst overall performance (Table 3).

Furthermore, our results demonstrate that, among the different influential factors, the vibration of the traction components had the greatest effect on the overall performance of the tubular scraper conveyors, followed by the material filling coefficient and the scraper space.

### 3.2. Validation Results

To validate the results obtained from the TOPSIS-based method, we used the measurement data supplied by the manufacturer for comparison. The results are summarized in Table 4. The ranking results of the three conveyors obtained from the TOPSIS-based method were highly consistent with those provided by the manufacturer.

## 4. Discussion

The overall performance of tubular scraper conveyors is affected by a variety of factors. To date, a reasonable evaluation method for the overall performance of tubular scraper conveyors has not been proposed. In this study, we used the TOPSIS method to establish an ideal model for the overall performance evaluation of tubular scraper conveyors based on the scraper space, material filling coefficient, and the dynamic load of the traction components, which are the primary factors that affect the overall performance of tubular scraper conveyors. To test the validity of our results, we compared the TOPSIS-based results with those provided by the manufacturer, which indicated that the TOPSIS-based results were consistent with those provided by the manufacturer. Although previous studies on tubular scraper conveyors using dynamic analyses and three-dimensional models have optimized machine parts, these studies failed to optimize the overall performance of tubular scraper conveyors based on human factors [6]. The application of the TOPSIS method reduces the subjective influence in the conveyor design. Moreover, this method can serve as an effective evaluation tool for the overall performance of tubular scraper conveyors. Furthermore, the results in this study indicate that the dynamic load of the traction components exhibits the largest effect on the overall performance, followed by the material filling coefficient and then the scraper space.

This study has a number of limitations. Fierce market competition makes data collection a challenging task. Consequently, we only considered three major factors that affect the overall performance of tubular scraper conveyors, namely, the scraper space, material filling coefficient, and the dynamic load of the traction components. Other factors, such as characteristics of the granular materials, the interspace between the scraper and the trough, and the thickness of the scraper, also affect the overall performance of tubular scraper conveyors. Therefore, additional associated data must be collected in the future. Furthermore, the TOPSIS-based method for evaluating the overall performance of tubular scraper conveyors can be improved. A more ideal and more rational mathematical model that better reflects the weights of different influencing factors on the overall performance is still needed and will benefit the design of energy-saving and highly efficient tubular scraper conveyors.

## Figures and Tables

**Table 1:** Attributes of the tubular scraper conveyors of the same type.

Conveyor	Scraper space (*t*)	Material filling coefficient (*η*)	Vibration coefficient of traction components (*k*)
A	2.7	3.1	4.2
B	2.6	3.0	4.4
C	2.5	3.2	4.3

**Table 2:** Field measurement results.

	Power consumption (kW)	Noise (dB)	Order
A	5.85	21.6	3
B	6.10	18.9	2
C	5.82	15.7	1

**Table 3:** The overall performance of the three tubular scraper conveyors evaluated using the TOPSIS-based method.

Target	*L*_*i*_^+^	*L*_*i*_^−^	*y*_*i*_	Order
A	0.0484	0.0323	0.4006	3
B	0.0354	0.0429	0.5481	2
C	0.0397	0.0463	0.5384	1

**Table 4:** Comparison between the field measurement data and the TOPSIS-based results of the overall performances of the three tubular scraper conveyors.

	Field measurement results			TOPSIS-based ordering results
	Power consumption (kW)	Noise (dB)	Order	
A	5.85	21.6	3	3
B	6.10	18.9	2	2
C	5.82	15.7	1	1
